# Updates in the skeletal and joint protective effects of tocotrienol: a mini review

**DOI:** 10.3389/fendo.2024.1417191

**Published:** 2024-06-21

**Authors:** Kok-Yong Chin

**Affiliations:** Department of Pharmacology, Faculty of Medicine, Universiti Kebangsaan Malaysia, Cheras, Malaysia

**Keywords:** bone, cartilage, osteoarthritis, osteoporosis, vitamin E

## Abstract

Osteoporosis and osteoarthritis continue to pose significant challenges to the aging population, with limited preventive options and pharmacological treatments often accompanied by side effects. Amidst ongoing efforts to discover new therapeutic agents, tocotrienols (TTs) have emerged as potential candidates. Derived from annatto bean and palm oil, TTs have demonstrated efficacy in improving skeletal and joint health in numerous animal models of bone loss and osteoarthritis. Mechanistic studies suggest that TTs exert their effects through antioxidant, anti-inflammatory, Wnt-suppressive, and mevalonate-modulating mechanisms in bone, as well as through self-repair mechanisms in chondrocytes. However, human clinical trials in this field remain scarce. In conclusion, TTs hold promise as agents for preventing osteoporosis and osteoarthritis, pending further evidence from human clinical trials.

## 1 Introduction

**Figure 1:**
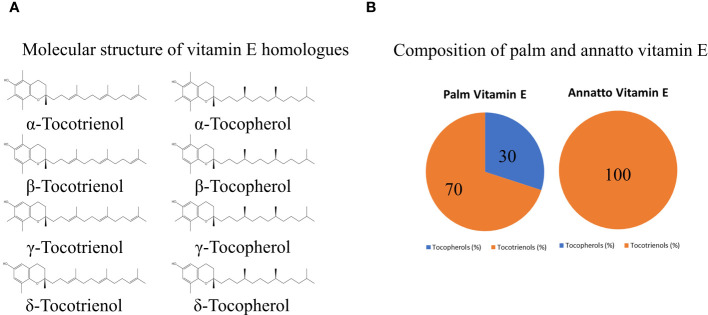
The molecular structure of vitamin E homologues **(A)** and exemplary composition of vitamin E from palm and annatto bean **(B)**.

Vitamin E encompasses a range of compounds falling into two primary families: tocopherols (TPs) and tocotrienols (TTs). Both families share a common molecular structure consisting of a chromanol ring and a long carbon tail. However, a key distinction lies in the carbon tail composition, with TTs featuring three unsaturated bonds while TPs have a saturated carbon tail. This structural variation confers unique biological properties to TTs not shared by TPs. Furthermore, each family can be subdivided into four homologues (α-, β-, γ-, and δ-) based on the position of the methyl group on the chromanol ring (1, 2). Natural sources, such as oil palm (~30% αTP, ~70% mixed TTs) (3), and annatto bean (negligible TP, ~10% γTT, ~90% δTT) (4), contain varying compositions of TTs and TPs (**Figure 1**). These differences in composition may influence interactions between vitamin E homologues and their biological activities within natural mixtures.

TTs or TT-enriched vitamin E fractions have demonstrated protective effects against bone and joint degeneration (5), which are particularly relevant in societies grappling with age-related health challenges. Osteoporosis and osteoarthritis are significant musculoskeletal conditions associated with aging, contributing substantially to healthcare burdens, with 16.6 million and 189.49 million disability-adjusted life-years, respectively, according to the latest Global Burden of Disease Survey in 2019 (6, 7). Despite lifestyle interventions, there remains a limited array of pharmacological preventive agents for both conditions (8, 9). TTs represent a promising avenue to address this gap by potentially serving as an effective preventive agent.

The skeletal effects of TTs have been extensively reviewed in previous studies (10–12). This review, however, aims to discuss recent advancements in establishing TTs as a therapeutic agent against osteoporosis and osteoarthritis. It will synthesize and evaluate recent evidence concerning the use of individual TTs or mixtures in preclinical models and human studies. Additionally, the review will outline future directions for research in this field.

## 2 Osteoporosis

Osteoporosis arises from an imbalance in bone remodeling, characterized by excessive bone resorption by osteoclasts and inadequate bone formation by osteoblasts. This imbalance leads to a net loss of bone mass, compromised microarchitecture, and reduced strength, consequently heightening the risk of fractures (13). The pathogenesis of osteoporosis involves various endogenous and exogenous factors, including sex hormone deficiency, excess glucocorticoids, and oxidative stress (14). Recent research indicates that increased fat mass may also negatively impact bone health by inducing inflammation and disrupting the hypothalamic-pituitary-adrenal axis (15).

### 2.1 Effects of TTs on osteoporosis

Studies on animals generally suggest that TT mixtures (60 mg/kg body weight) can improve bone structural histomorphometry in normal rats and those with induced bone loss from factors such as estrogen deficiency, testosterone deficiency, nicotine, and glucocorticoids (**Table 1**). These improvements are attributed to increased osteoblast numbers and bone formation activities, as well as decreased osteoclast numbers and bone resorption activities (16, 19, 22, 31, 34). Recent investigations employing micro-computed tomography have explored the effects of TT mixtures on bone microstructure. Annatto TT significantly improved some trabecular microstructural indices in rats subjected to orchidectomy and androgen deprivation therapy (16, 20). Additionally, studies showed that annatto and palm TTs preserved trabecular microstructure in rats fed a high-fat, high-carbohydrate diet (25, 35).

**Table 1:** Effects of tocotrienol on bone health indices in several representative animal model of osteoporosis.

Researchers	Animals	Model of bone loss	Treatment	Outcomes (improvement vs negative control)	
Chin & Ima (2014) (16) Chin et al. (2014) (17) Chin et al. (2016) (18)	3-month-old Sprague- Dawley male rats	Androgen deficiency Bilateral orchidectomy	Annatto TT (10% γ-TT, 90% δ-TT) 60 mg/kg/day for 2 months	µCT	↓ TbSp
				Structural histomorphometry	↑ BV/TV, ↑ TbN, ↓ TbSp
				Dynamic histomorphometry	↑ dLS/BS, ↓ sLS/BS
				Cellular histomorphometry	↑ ObS/BS, ↓ OcS/BS, ↓ ES/BS, ↑ OS/BS, ↑ OV/BV
				Bone gene expression	↑ ALPL, ↑ COL1α1, ↑ SPP1, ↓ RANKL, ↓ PPARG
				Mineral	↓ serum total calcium, ↑ bone total calcium
				Biomechanical strength	No difference
				Bone remodelling markers	No difference
Mohamad et al. (2018) (19) Mohamad et al. (2018) (20)	3-month-old Sprague-Dawley male rats	Androgen deficiency Buserelin (75 μg/kg/day)	Annatto TT (10% γ-TT, 90% δ-TT) 60 mg/kg/day for 2 months	µCT	↑ BV/TV ↓ TbSp, ↑ CtTh
				Structural histomorphometry	↑ BV/TV, ↑ TbTh
				Dynamic histomorphometry	↑ sLS/BS, ↑ dLS/BS (61)
				Cellular histomorphometry	↑ ObN/BS
				Mineral	↑ Bone calcium content
				Biomechanical strength	↑ Max load, ↑ strain, ↑ elastic modulus
				Bone remodelling markers	No difference
Mohamad et al. (2021) (22) Mohamad et al. (2021) (23)	8-month-old Sprague-Dawley female rats	Estrogen deficiency Bilateral ovariectomy, followed by 2 months of resting	Emulsified and non-emulsified Annatto TT (10% γ-TT, 90% δ-TT) 60 mg/kg/day for 2 months	µCT	Both: ↑ CtTh; AnTT-SEEDS: ↑ TbTh, ↑ TbN
				Dynamic histomorphometry	Both: ↓ sLS/BS, ↑ MAR; AnTT-SEDDS: ↑ dLS/BS, ↑ BFR
				Cellular histomorphometry	Both: ↑ ObS/BS; AnTT-SEDDS: ↑ OV/BV
				Mineral	Both: ↑ Bone calcium content
				Biomechanical strength	Both: ↑ load, ↑ stress, ↑ Young’s Modulus; AnTT-SEEDS: ↑ Stiffness
				Bone remodelling markers	Both: ↓ SOST; AnTT-SEDDS: ↓ RANKL/OPG ratio Both: ↑ SOD, ↑ GPX;
				Redox status	AnTT-SEEDS: ↓ malondialdehyde
Ekeuku et al. (2020) (24)	3-month-old Sprague-Dawley female rats	Estrogen deficiency + osteoarthritis Bilateral ovariectomy (osteoporosis) Monosodium iodoacetate (osteoarthritis) *bone health indices were assessed on the leg without osteoarthritis	Emulsified (α-TP 6.9%, α-TT 6.6%, β-TT 1.1%, γ-TT 9.4%, δ-TT 3.2%) or non-emulsified palm TT mixture (α-TP 10.3%, α-TT 12.9%, β-TT 2.0%, γ-TT 19.8%, δ-TT 6.3%) 100 mg/kg for 2 months	BMD	No difference
				Structural histomorphometry	↑ BV/TV, ↑ TbN (NEPT)
				Dynamic histomorphometry	No difference
				Cellular histomorphometry	No difference
				Mineral	No difference
				Biomechanical strength	EPT: ↓ strain, ↓ displacement, ↑ stiffness, ↑ Young’s modulus
				Bone remodelling markers	No difference
Wong et al. (2018) (25) Wong et al. (2018) (26) Wong et al. (2019) (27)	3-month-old Wistar male rats	Metabolic syndrome High fat high carbohydrate diet for 20 weeks	Palm TT mixture (21.9% α-TP, 24.7% α-TT, 4.5% β-TT, 36.9% γ-TT, and 12.0% δ-TT) 60 and 100 mg/kg/day for 2 months) – treatment started from week 8 until week 20	BMD	No difference
				µCT	Both doses: ↑ BV/TV, ↓ TbSp. ↓ SMI, ↑ TbN
				Structural histomorphometry	Both doses: ↑ BV/TV, ↑ TbTh
				Dynamic histomorphometry	60 mg/kg/day: ↓ sLS/bS, ↑ MAR
				Cellular histomorphometry	Both doses: ↑ ObS/BS, ↑ OS/BS
				Bone calcium content	No difference
				Biomechanical strength	Both doses: ↑ load, ↑ Young’s modulus; 60 mg/kg/day: ↑ stiffness
				Bone remodelling markers	Both doses: ↓ RANKL, ↓ FGF-23; 100 mg/kg/day: ↓ SOST, ↓ DKK1
				Circulating inflammation markers	Both doses: ↓ IL-1α; ↓ IL-6
Wong et al. (2018) (26) Wong et al. (2019) (27)	3-month-old Wistar male rats	Metabolic syndrome High fat high carbohydrate diet for 20 weeks	Annatto TT (10% γ-TT, 90% δ-TT) 60 or 100 mg/kg/day) – treatment started from week 8 until week 20	BMD	No difference
				µCT	Both doses: ↑ BV/TV, ↓ TbSp. ↓ SMI, ↑ TbN, ↑ Conn.D
				Structural histomorphometry	Both doses: ↑ BV/TV, ↑ TbTh
				Dynamic histomorphometry	60 mg/kg/day: ↓ sLS/bS
				Cellular histomorphometry	Both doses: ↑ ObS/BS
				Bone calcium content	No difference
				Biomechanical strength	100 mg/kg/day: ↑ load, ↑ strain, ↑ Young’s modulus
				Bone remodelling markers	Both doses: ↓ RANKL, ↓ FGF-23; 100 mg/kg/day: ↓ SOST, ↓ DKK1
				Circulating inflammation markers	Both doses: ↓ IL-1α; ↓ IL-6
Chin et al. (2020) (28)	3-month-old male Sprague-Dawley rats	Proton pump inhibitor Pantoprazole (3 mg/kg/day for 60 days)	Annatto TT (10% γ-TT, 90% δ-TT) 60 mg/kg/day for 60 days	Gross anatomy	No change in femoral length, diameter, weight
				µCT	↑ BV/TV, ↑ TbN, ↓ TbSp, ↑ TbTh
				Cellular histomorphometry	No difference
				Biomechanical strength	No difference
Ima-Nirwana & Fakhrurazi (2002) (29)	3-month-old male Wistar rats	Adrenalectomy + Glucocorticoids (dexamethasone 120 µg/kg/day or deoxycorticosterone 2400 µg/kg/day) started 1 week after adrenalectomy for 8 weeks	Palm TT mixture (24.82% α-TP, 20.73% α-TT, 26,68% γ-TT and 13.32% δ-TT) 60 mg/kg – started 1 week after adrenalectomy for 8 weeks	BMD	No difference but palm TT sustained age-related BMD ↑
				Gross anatomy	No difference in femoral length
				Bone calcium content	No difference
Ima-Nirwana & Suhaniza (2004) (30)	4-month-old male Sprague-Dawley rats	Adrenalectomy + Glucocorticoids (dexamethasone 120 or 240 µg/kg/day) started 2 weeks after adrenalectomy for 8 weeks	γ-TT or α-TP at 60 mg/kg – started 2 weeks after adrenalectomy for 8 weeks	BMD	No difference
				Bone calcium content	Both: ↑ in lumbar 4
Hermizi et al. (2009) (31)	3-month-old male Sprague-Dawley rats	Nicotine cessation Nicotine (7 mg/kg) 6 days a week for 2 months.	Palm TT mixture (α-TT 43%, γ-TT 31%, δ-TT 14%, and other oils 12%), γ-TT & α-TP at 60 mg/kg/day - treatment for 2 months after nicotine cessation	Structural histomorphometry	All treatments: ↑ BV/TV, ↑ TbTh; α-TP: ↑ TbN
				Dynamic histomorphometry	All treatments: ↓ sLS/BS, ↑ MAR, ↑ BFR/BS
				Cellular histomorphometry	All treatments: ↓ OcS/BS, ↓ ES/BS
Norazlina et al. (2007) (32)	3-month-old male Sprague-Dawley rats	Nicotine cessation Nicotine (7 mg/kg) 6 days a week for 2 months.	Palm TT mixture or α-TP 60 mg/kg/day – treatment for 2 months after nicotine cessation	Bone mineral content	No difference
				Circulating inflammatory cytokines	↓ IL-6
Abukhadir et al. (2012) (33)	3-month-old male Sprague-Dawley rats	Nicotine cessation Nicotine (7 mg/kg) 6 days a week for 2 months.	Palm TT mixture 60 mg/kg/day (composition not disclosed) – treatment for 2 months after nicotine cessation	Bone gene expression	↑ BMP-2, ↑ OSX, ↑ RUNX2

ALPL, alkaline phosphatase; AnTT-SEEDS, annatto tocotrienol with self-emulsifying system; BFR, bone formation rate; BMD, bone mineral density; BMP-2, bone morphogenetic protein-2; BS, bone surface; BV/TV, bone volume over tissue volume; COL1α1, collagen 1 alpha 1; Conn.D, connectivity density; CtTh, cortical thickness; DKK1, Dickkopf-1; dLS/BS, double-labelled surface; EPT, emulsified palm tocotrienol; ES/BS, eroded surface; FGF-23, fibroblast growth factor 23; GPX, glutathione peroxidase; IL, interleukin; MAR, mineralizing surface; NEPT, non-emulsified palm tocotrienol; PPARG, peroxisome proliferator activated receptor gamma; ObN/BS, osteoblast number; ObS/BS, osteoblast surface; OcS/BS, osteoclast surface; OPG, osteoprotegerin; OS/BS, osteoid surface; OSX, osterix; OV/BV, osteoid volume; RANKL, receptor activator of nuclear factor kappa beta; RUNX2, runt related factor 2; sLS/BS, single-labelled surface; SMI, structural model index; SOD, superoxide dismutase; SOST, sclerostin; SPP1, osteopontin; TbN, trabecular number, TbSp, trabecular separation; TbTh, trabecular thickness; TP, tocopherol; TT, tocotrienol; µCT, micro-computed tomography; ↑ increase; ↓ decrease.

However, the effects of TTs on bone mineral density, calcium content, and biomechanical strength assessed through various methods such as dual-energy X-ray absorptiometry and atomic absorption spectrophotometry, are less consistent. These inconsistencies may be attributed to variations in the models of bone loss utilized and the duration of treatment (10).

There is a caveat in the previous studies, whereby most have utilized sexually mature rats aged three months old as a model. While commonly employed in osteoporosis research due to their cost-effectiveness and accessibility, these rats may not fully replicate osteoporosis in humans. Unlike humans, rats experience continuous longitudinal skeletal growth because they do not undergo growth plate closure (36). Thus, the use of growing rat might represent a stunted bone growth model rather than an osteoporosis model due to ongoing bone modeling processes.

Furthermore, the bone protective effects of annatto TT have been investigated in postmenopausal women with osteopenia. A 12-week study involving supplementation with 430 mg or 860 mg of annatto TT showed significant decreases in urine N-terminal telopeptide (NTX) levels (a bone resorption marker), circulating receptor activator of nuclear factor kappa-B ligand (RANKL) levels, and RANKL/osteoprotegerin ratios, as well as an increase in bone alkaline phosphatase/NTX ratio (a bone formation marker) compared to a placebo control group (37). However, long-term studies examining the effects of annatto TT on bone mineral density have yet to be conducted.

Fragility fractures are the consequence of untreated osteoporosis (38). TT’s potential in facilitating fracture healing has been explored in several animal models, where TT administration, alone or in combination with other agents, has been shown to promote callus formation and increase the biochemical strength of the fracture site (39, 40). However, clinical trial evidence regarding TT’s effects in this aspect remains lacking.

### 2.2 Bone protective mechanisms of TT

The skeletal protective mechanism of TTs has traditionally been attributed to their antioxidant and anti-inflammatory activities. Numerous studies have shown that TTs reduce systemic and skeletal oxidative stress markers in rats with bone loss (23, 41, 42). *In vitro* exposure of osteoblasts to TTs has also demonstrated a mitigation of negative oxidant effects (43, 44). Moreover, supplementation with TTs has led to a reduction in circulating inflammatory markers, such as interleukin (IL)-1 and IL-6, in animals with induced bone loss (35, 45). In women with osteopenia, metabolic study revealed increased lysophospholipids, but decreased acylcarnitines and catabolites of tryptophan and steroids, indicating suppression of inflammation and oxidative stress (46). However, direct evidence on how TTs influence immune cell populations and activities to achieve bone protective effects is lacking. Recent studies have proposed that TTs may regulate gut microbiota (47), suggesting a potential influence on the gut-bone axis to achieve their anti-osteoporosis potential.

TTs have been shown to promote osteoblast differentiation and bone formation activity in both two- and three-dimensional cultured systems (48–50). Among the homologues, γ and δ-TT were found to be most pro-osteogenic (48). Alpha-TT has been found to suppress osteoclast formation from bone marrow cell and osteoblast cocultures by inhibiting c-FOS expression, attributed to the inhibition of ERK and NF-κB activation (51). Multiple animal studies in ovariectomized mice have demonstrated that TT supplementation reduces RANKL levels and, consequently, the RANKL/OPG ratio, reducing osteoclastogenesis and bone resorption (22, 24). This observation has been supported by randomized controlled trials (37).

Research on the effects of TTs on osteocyte function is actively underway. Osteocytes, acting as bone remodeling mediators and mechanical load sensors, play a pivotal role in regulating bone metabolism. They secrete RANKL and OPG to influence the bone remodeling process, as well as inhibitors of the Wnt signaling pathway critical for osteoblastogenesis, such as Dickkopf-1 (DKK1) and sclerostin (SOST) (52). Studies have shown that TT supplementation increases skeletal mRNA expression of β-catenin, the central transcription factor in the Wnt pathway, in orchidectomized rats (16). Subsequent studies have demonstrated reduced skeletal levels of SOST and DKK1 proteins upon TT supplementation in rats with estrogen deficiency and metabolic syndrome (22, 27). *In vitro* studies using pre-osteoblasts have shown that δ-TT suppresses critical signaling molecules in the Wnt pathway, stimulating cell proliferation and differentiation (53). Wnt signaling has also been shown to mediate the action of δ-TT in promoting osteoblast migration (54).

The mevalonate pathway, crucial for protein prenylation process of GTPase critical for cellular function, is inhibited by TTs via a mechanism distinct from statins, particularly through post-translational suppression of 3-hydroxy-3-methylglutaryl-coenzyme A reductase (HMGR) (55). Exposure of MC3T3-E1 pre-osteoblast cells to δ-TT resulted in decreased HMGR protein expression and increased differentiation markers. Additionally, annatto TT decreased HMGR mRNA expression while increasing RhoA activity in MC3T3-E1 pre-osteoblast cells, promoting their differentiation (49). Co-supplementation of mevalonate reversed the bone protective effects of δ-TT in ovariectomized mice, indicating the importance of the mevalonate pathway in TT-mediated bone protection (56).

**Figure 2:**
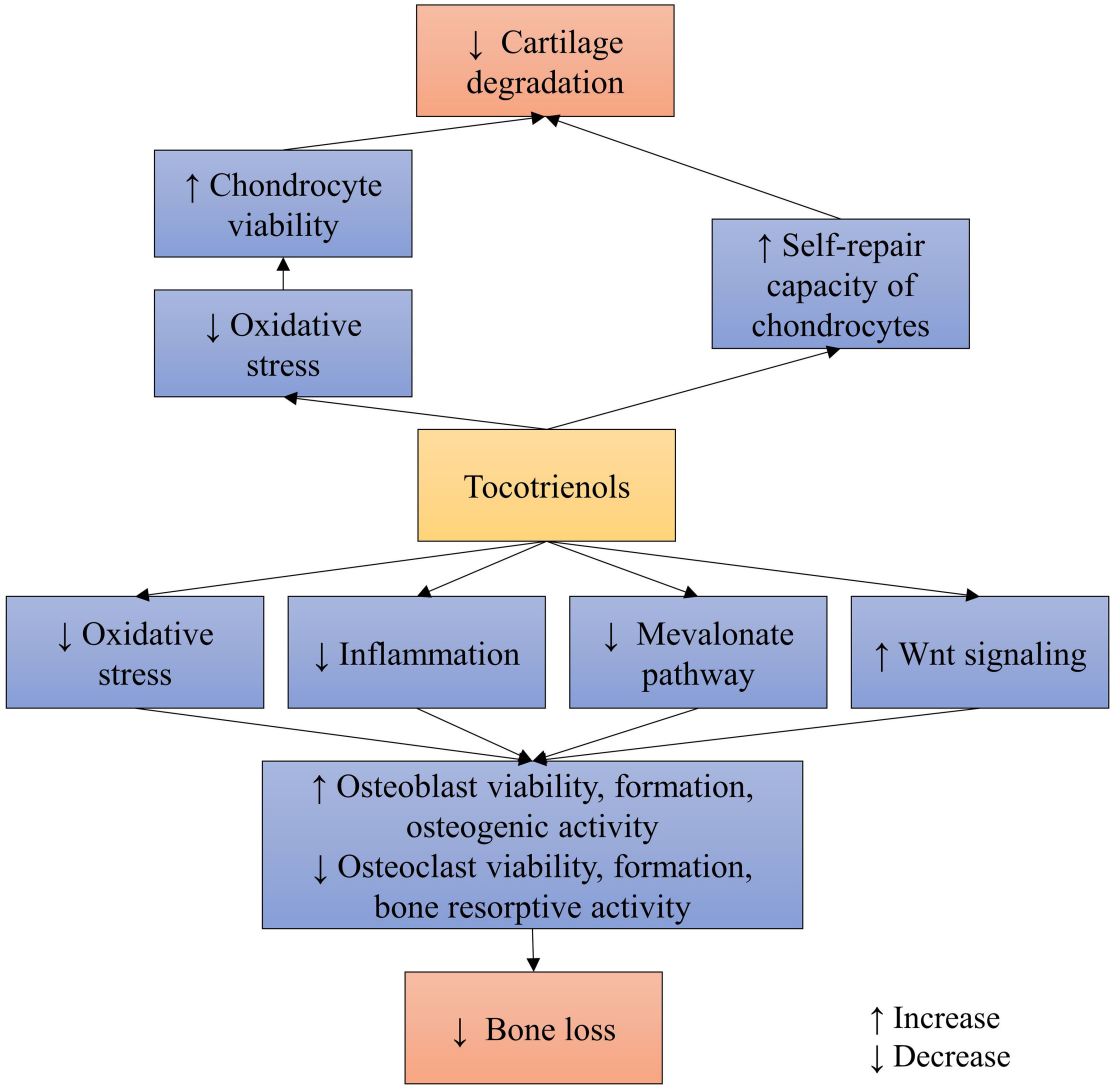
The protective mechanism of TT on bone and cartilage.

In summary, TTs can promote bone health directly through various signaling pathways (**Figure 2**), beyond their conventional antioxidant and anti-inflammatory actions. However, more in depth studies are needed to illustrate the signaling cascades involved for better understanding of TTs’ actions.

## 3 Osteoarthritis

Osteoarthritis is characterized by significant articular cartilage loss, subchondral bone changes, and osteophyte formation, leading to joint pain, swelling, and stiffness (57). Traditionally categorized as non-inflammatory arthritis, recent research has underscored the substantial involvement of inflammation in the local joint environment (58). Wear-and-tear particles generated from cartilage due to accumulated mechanical damage attract macrophages into the joint space. These particles also prompt chondrocytes to release metalloproteinases, exacerbating cartilage breakdown. Moreover, these particles serve as damage-associated molecular patterns, activating toll-like receptor signaling in synoviocytes and chondrocytes, thereby increasing the synthesis of pro-inflammatory cytokines. Initially attempting to compensate for cartilage loss, chondrocytes become hypertrophied before eventually becoming senescent and losing functionality (59).

### 3.1 Effects of TT on osteoarthritis

The effects of annatto TT and palm TT have been assessed in rats induced with osteoarthritis using intra-articular monosodium iodoacetate (MIA) injection. MIA exposure, acting as a glycolysis inhibitor, induces mitochondrial stress and chondrocyte death (60), producing structural and pain response in rodents akin to osteoarthritis in humans (61, 62). An initial pilot study demonstrated that at 100 mg/kg, annatto TT prevented degenerative joint changes and reduced circulating joint degradation markers, cartilage oligomeric matrix protein, and hyaluronan (63). At a higher dose (150 mg/kg), annatto TT decreased osteocalcin levels and osteoclast surface at the subchondral bone, suggesting suppression of bone remodeling (63). Subsequent analyses evaluated the effects of palm TT mixture alone or in combination with glucosamine sulfate in MIA-induced osteoarthritis in rats (64). Palm TT alone or combined with glucosamine sulfate effectively suppressed circulating cartilage oligomeric matrix protein levels but did not significantly alter Mankin’s scores of the joint. Only combination therapy increased the retention time of rats on inverted mesh wire, a surrogate measure of grip strength, albeit not specific to the affected hindlimb (64). Variations in the anti-osteoarthritis potential between annatto and palm tocotrienol mixtures may stem from differences in tocotrienol homologue composition.

Given the greater prevalence of osteoarthritis among women compared to men, the effects of palm tocotrienol were evaluated in ovariectomized rats (24). After bilateral ovariectomy, rats rested for one month before receiving intra-articular MIA injections. Prophylactic treatment with palm TT mixtures (two formulations: 100 mg/kg unformulated or 50% less TT in emulsified version) commenced immediately post-ovariectomy. Both palm TT formulations prevented joint swelling and cartilage degradation, with only emulsified palm TT preventing grip strength decline. Additionally, both palm TT formulations preserved subchondral bone volume, but only emulsified palm TT significantly increased bone formation rate, while unformulated palm TT decreased osteoclast surface on subchondral bone (24).

Furthermore, the efficacy of palm TT mixture (400 mg/day) on physical function and pain in osteoarthritis patients was compared with glucosamine sulfate (1500 mg/day) over 6 months (65). No significant difference in Western Ontario and McMaster Universities osteoarthritis index (WOMAC) or visual analogue scale scores was observed between groups. However, the palm TT-supplemented group exhibited significantly lower malondialdehyde levels compared to the glucosamine sulfate-supplemented group (65).

### 3.2 Joint protective mechanisms of TT

The protective mechanisms of annatto TT and palm TT on chondrocytes have been explored in an *in vitro* study utilizing SW1353 chondrocytes exposed to MIA (66). The findings suggest that TTs mitigate oxidative stress in chondrocytes subjected to MIA-induced damage. Particularly noteworthy is the observation that concurrent exposure to annatto TT and MIA may activate a self-repair mechanism in the SW1353 chondrocyte cell line. This was evidenced by a significant increase in SRY-Related HMG Box Gene 9, collagen 2α1, and aggrecan protein levels, along with a decrease in A disintegrin and metalloproteinase with thrombospondin motifs 4 levels (66) (**Figure 2**). However, further mechanistic studies are warranted to elucidate the precise mechanisms through which TTs protect cartilage in osteoarthritis.

## 4 Future directions

Our body preferentially absorbs α-TP over other vitamin E homologues due to the presence of α-TP transport protein (67). Consequently, the presence of α-TP in the diet or vitamin E mixture could potentially interfere with the absorption of TTs (68). Several attempts have been made to enhance the absorption of TTs. One such approach is the utilization of self-emulsifying delivery systems (SEDDS), which aims to increase the bioavailability of TTs by partitioning them into smaller micelles with a larger surface-to-body ratio to facilitate intestinal absorption (21). However, a study by Mohamed et al. (23) showed that while SEDDS increased serum TT levels by fourfold, it did not significantly improve the efficacy of TT in preventing bone loss (23). These findings suggest a potential threshold for the skeletal effects of TT. Similarly, in the study by Shen et al. (37), women with osteopenia who received two doses of annatto tocotrienol (430 mg/day versus 860 mg/day) did not exhibit significant differences in bone remodeling markers (37). Subsequently, Ekeuku et al. (24) compared the bone and joint protective effects of an unformulated palm TT mixture with a commercialized emulsified palm TT mixture containing 50% less TT. They found similar total circulating vitamin E levels and skeletal and joint effects in ovariectomized rats fed with both palm TT formulations (24). These findings suggest that SEDDS could potentially reduce the amount of TTs required to achieve therapeutic effects.

Clinical evidence regarding the musculoskeletal side effects of TTs has been limited thus far. The clinical trial conducted by Shen et al. (37) on women with osteopenia demonstrated the benefits of annatto TT in suppressing bone resorption (37), but whether it can slow down the decline of bone mineral density remains uncertain. Moreover, since all groups in the trials were supplemented with calcium and vitamin D, it is plausible that any differences in skeletal parameters between TT-supplemented and unsupplemented groups may have been minimized. Furthermore, only one trial has been conducted thus far on the effects of palm TT on osteoarthritis. Haflah et al. (65) compared the effects of TT with glucosamine sulfate in improving visual analogue scale scores, which measure pain intensity, as well as WOMAC scores, which assess joint pain, stiffness, and function (65). The lack of difference between the two groups suggests that palm TT may be as effective as glucosamine sulfate in improving joint function and reducing joint pain. However, no clinical studies have been conducted to investigate the structural changes in the joint caused by TT supplementation.

In summary, further research is needed, including exploration of innovative delivery systems and conducting more clinical trials, to fully understand the potential benefits and mechanisms of action of TTs in musculoskeletal health.

## 5 Conclusion

The accumulated preclinical evidence thus far strongly suggests that TTs could protect bone health in individuals at risk of osteoporosis. However, only one clinical trial has validated the skeletal protective effects of TT. Similarly, a series of studies have been conducted to investigate the promising effects of TT in preventing osteoarthritis, but only one study validated the preclinical findings using questionnaire-based assessment tools. On that note, we need more well-planned randomized controlled trials to translate the putative benefits of TT to patients with osteoporosis and osteoarthritis.

## Author contributions

KC: Conceptualization, Funding acquisition, Investigation, Visualization, Writing – original draft.
